# Effects of Calcium Ions on the Antimicrobial Activity of Gramicidin A

**DOI:** 10.3390/biom12121799

**Published:** 2022-12-01

**Authors:** Shang-Ting Fang, Shu-Hsiang Huang, Chin-Hao Yang, Jen-Wen Liou, Hemalatha Mani, Yi-Cheng Chen

**Affiliations:** 1 Department of Medicine, MacKay Medical College, New Taipei City 252, Taiwan; 2 Department of Biochemistry, School of Medicine, Tzu Chi University, Hualien City 97004, Taiwan

**Keywords:** gramicidin A, antimicrobial activity, Ca^2+^ ions, hydroxyl free radical, NAD^+^/NADH ratio

## Abstract

Gramicidin A (gA) is a linear antimicrobial peptide that can form a channel and specifically conduct monovalent cations such as H^+^ across the lipid membrane. The antimicrobial activity of gA is associated with the formation of hydroxyl free radicals and the imbalance of NADH metabolism, possibly a consequence caused by the conductance of cations. The ion conductivity of gramicidin A can be blocked by Ca^2+^ ions. However, the effect of Ca^2+^ ions on the antimicrobial activity of gA is unclear. To unveil the role of Ca^2+^ ions, we examined the effect of Ca^2+^ ions on the antimicrobial activity of gramicidin A against *Staphylococcus aureus* (*S. aureus*). Results showed that the antimicrobial mechanism of gA and antimicrobial activity by Ca^2+^ ions are concentration-dependent. At the low gA concentration (≤1 μM), the antimicrobial mechanism of gA is mainly associated with the hydroxyl free radical formation and NADH metabolic imbalance. Under this mode, Ca^2+^ ions can significantly inhibit the hydroxyl free radical formation and NADH metabolic imbalance. On the other hand, at high gA concentration (≥5 μM), gramicidin A acts more likely as a detergent. Gramicidin A not only causes an increase in hydroxyl free radical levels and NAD^+^/NADH ratios but also induces the destruction of the lipid membrane composition. At this condition, Ca^2+^ ions can no longer reduce the gA antimicrobial activity but rather enhance the bacterial killing ability of gramicidin A.

## 1. Introduction

Gramicidin is a linear pentadecapeptide with alternating L- and D-amino acids [1,2,3]. There are three isoforms, gramicidin A (gA), gramicidin B (gB), and gramicidin C (gC), which are different at the residue 11, Trp for gA, Phe for gB and Tyr for gC, respectively [2,3]. The alternating D- and L- sequence makes gramicidin adopt an unusual β^6.3^-sheet type of helix [3,4]. Gramicidin can adopt a number of different β-helical conformations, including double helices and single-strand helical monomers [4,5,6,7,8]. The double helix is two monomers interwound, similar to DNA double helices [4,6,8]. The double helix can be either parallel or antiparallel, left- or right-handed double-stranded helices in organic solvents [6,7,8,9]. Unlike the double helix, the single-stranded helical monomer is usually right-handed and forms in polar organic solvents such as trifluoroethanol (TFE) [4,10,11,12]. Two single-strand helical monomers can form an N-head-to-N-head dimer in a lipid environment [4,11,12]. The function of the N-head-to-N-head dimer has been proposed to be a channel for specifically translocating the alkali cations and H^+^ across lipid membranes [4,12,13,14].

Gramicidin A has antibiotic and antimalarial activities [15,16]. Two mechanisms have been associated with antimicrobial activity [17,18]. The first mechanism associated with the gramicidin A antimicrobial activity is to disrupt the cell membrane composition [17]. The second mechanism is associated with the formation of free radicals and the disruption of NAD^+^/NADH synthesis [18].

The antimicrobial activity is dependent on the structural state of gramicidin A [19,20]. Two previous studies have shown that the antimicrobial activity of gramicidin A is linked with the formation of ion channels [19,20]. By synthesizing several lactam-bridged gramicidin analogs, Mao and his colleagues showed that the gramicidin analogs with channel form retain antimicrobial activity [20]. In an alternated approach by the Arndt group, they synthesized several gramicidin A analogs with the thiol group at different positions [19]. These gramicidin A analogs formed stable conformers in membrane lipids. Arndt and his co-worker demonstrated that only the gramicidin A analogs with tail-to-tail antiparallel single strand β^6.3^-helix mediated antimicrobial and antimalarial activities [19]. In a recent study, we showed that calcium ions could dissociate double helix into single-strand helical monomer [21]. In this study, we further demonstrated that the single-strand helical monomer has the highest antimicrobial activity compared to the double helical dimer [21]. All these studies indicated that the gramicidin channel, instead of the gramicidin double helical dimer, is likely to be the active antimicrobial form. 

The gramicidin A channel can specifically conduct monovalent cations such as Li^+^, Na^+^, and K^+^ cross-membrane lipids [12,13,14]. The ion conductivity of gramicidin A can be blocked by Ca^2+^ ions [22,23]. However, the correlation between antimicrobial activity and Ca^2+^ ions has remained unclear. Therefore, in the present study, we investigated the effect of calcium cations on the antimicrobial activity of gramicidin A. Our results showed that the antimicrobial mechanism of gA is highly dependent on its concentration. The antimicrobial mechanism is mainly linked with increased hydroxyl free radical and NAD^+^/NADH ratios at low gA concentrations, while gramicidin A may act like a detergent and cause bacterial death through the disruption of membrane lipid composition. Ca^2+^ ions can only effectively inhibit the antimicrobial activity of gramicidin A at low gA concentrations through the inhibition of the antimicrobial activity of gramicidin A is associated with hydroxyl free radical formation and NADH metabolic imbalance. Due to the different antimicrobial mechanisms induced by the high concentration of gA, Ca^2+^ ions cannot inhibit but enhance the antimicrobial activity of gramicidin A. 

## 2. Material and Methods

### 2.1. Materials

Gramicidin A was purchased from Merck (Darmstadt, Germany). Calcium chloride, magnesium chloride, strontium chloride, Luria–Bertani (LB) broth, and trifluoroethanol were purchased from Sigma-Aldrich (St. Louis, MO, USA). 30-(p-hydroxyphenyl) fluorescein was purchased from Invitrogen (Thermo Fisher Scientific, Waltham, MA, USA). NAD/NADH Assay kit (ab176723) was purchased from Abcam PLC (Cambridge, UK). All other chemicals were reagent grade and used without further purification. 

### 2.2. Bacterial Growth Conditions

*Staphylococcus aureus* (*S. aureus*) (ATCC-25923) was purchased from Bioresource Collection and Research Center (BCRC), Taiwan. Twenty-five mL of *S. aureus* was grown in LB broth medium in a 250 mL flask at 37 °C overnight. This overnight *S. aureus* culture was diluted to OD600 = 0.1 with LB medium. This *S. aureus* was then grown to lag phase (OD600 = 0.1–0.4), exponential phase (OD600 = 0.4–0.9), and stationary phase (OD600 > 0.9). The stock solution of gramicidin A was prepared in a final concentration of 100 μM in trifluoroethanol. At the designated phase, the *S. aureus* samples treated with or without gramicidin A and cations with the designed concentration were incubated at 37 °C. The optical density at a wavelength of 600 nm (OD600) was used to determine the growth curve of *S. aureus* using a microplate reader (FlexStation 3, MD, San Jose, CA, USA) every 30 min.

### 2.3. Reactive Oxygen Species (ROS) Assay

To detect hydroxyl radical formation, the fluorescent reporter dye 30-(p-hydroxyphenyl) fluorescein (5 mM) was used. In all experiments, *S. aureus* samples at exponential phase treated with or without gA or cations were taken every hour for 3 h. The fluorescence intensity at an emission wavelength of 545 nm with an excitation wavelength of 488 nm was used to determine the related fold of hydroxyl radical using a microplate reader (FlexStation 3, MD, San Jose, CA, USA).

### 2.4. NAD/NADH Assay

The NAD^+^/NADH ratio was measured using an NAD^+^/NADH assay kit (Abcam, Waltham, MA, USA). The detailed experiments were performed according to the procedures provided by the kit. *S. aureus* treated with or without gA and cations incubated at 0, 1, 2, and 3 h were harvested and centrifugated at 10,000× *g* for 15 min at 4 °C. The cells were resuspended in a lysis buffer to a final concentration of 10^7^–10^8^ cells/mL. The resuspended cells were incubated at room temperature for 15 min and centrifugated at 2500 rpm for 5 min. The supernatant was transferred to a new tube and kept on ice. 

For NADH extraction, twenty-five μL of the sample with or without gA and cations was added to a well in a 96-well plate. Then, twenty-five μL of NADH extraction buffer was added to the well containing the sample and incubated for a further 15 min at 37 °C. After incubation, twenty-five μL of NAD^+^ extraction buffer was added to neutralize NADH extraction. Similar procedures were performed for NAD extraction, except that NAD extraction buffer was used to extract NAD, and NADH extraction buffer was used to neutralize the reaction. For the NAD^+^/NADH ratio, seventy-five μL of NADH reaction enzyme mixture was added into each well and incubated at room temperature for 2 h. The related concentration of NAD or NADH was measured using a fluorescence microplate reader (FlexStation 3, MD, San Jose, CA, USA) with an excitation wavelength of 540 nm and emission wavelength of 590 nm.

### 2.5. Atomic Force Microscopy (AFM)

All images were collected using an AFM (Nanowizard^TM^, JPK instruments, Berlin, Germany) installed on an inverted optical microscope (Nikon Corporation, Tokyo, Japan). The AFM probes used were oxidize-sharpened silicon nitride probes (OMCL-TR400PB-1, Olympus, Tokyo, Japan) with a spring constant of 0.02 N/m. Ten microliters of samples treated with or without gramicidin A or Ca^2+^ ions at the designed concentration were placed on a cleaved mica disc (Ted Pella Inc., Redding, CA, USA) for 20–40 min. The mica disc was then rinsed twice with distilled water and dried for 24 h. The dried samples were imaged in an AFM operating in a contact mode. Images were acquired at a scanning rate of 1–2 Hz and a resolution of 512 × 512 pixels. Image processing and analysis were performed using SPM software v. 3.16 (Nanowizard^TM^, JPK instruments, Berlin, Germany, Germany).

### 2.6. Statistical Analysis

Statistical analyses were done using the one-way ANOVA with Original 6.0 software. Data are expressed as mean ± standard deviation. *p*-value ≤ 0.05 was considered statistically significant.

## 3. Results

### 3.1. Verification of the Antimicrobial Effect and Mechanism of Gramicidin A 

**Figure 1:**
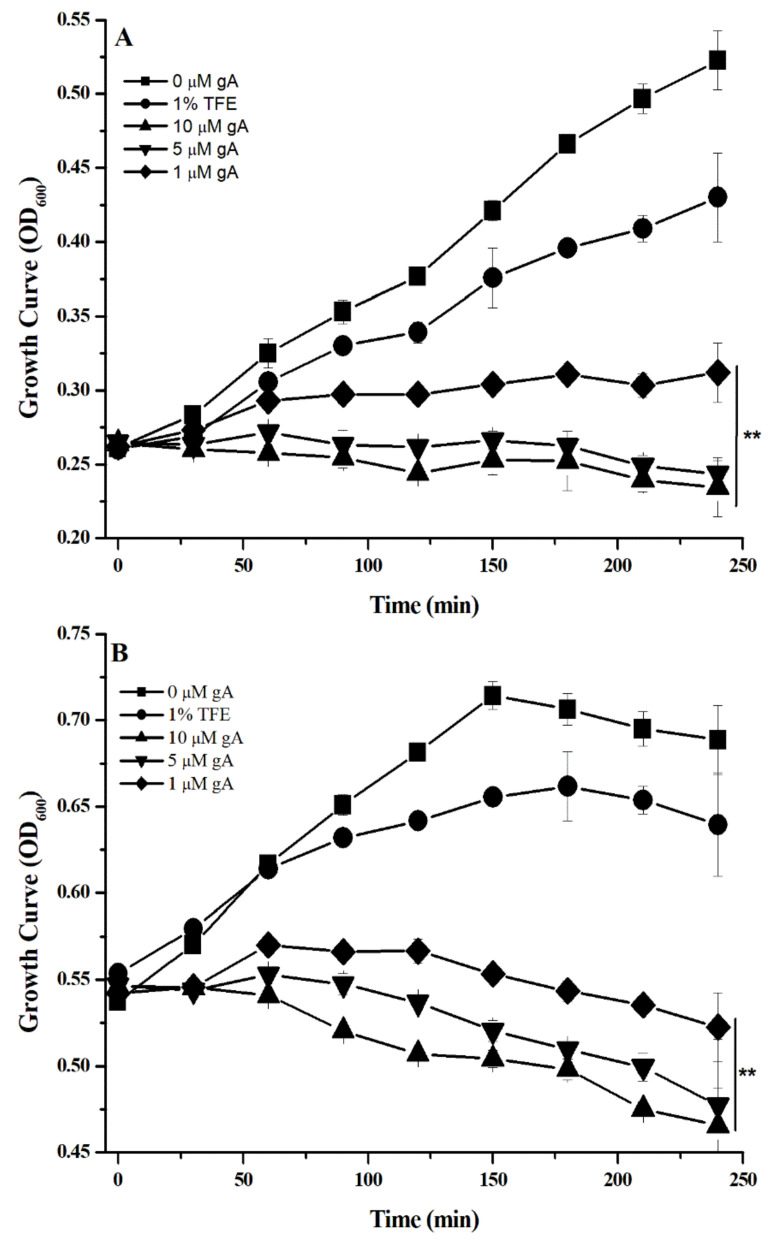

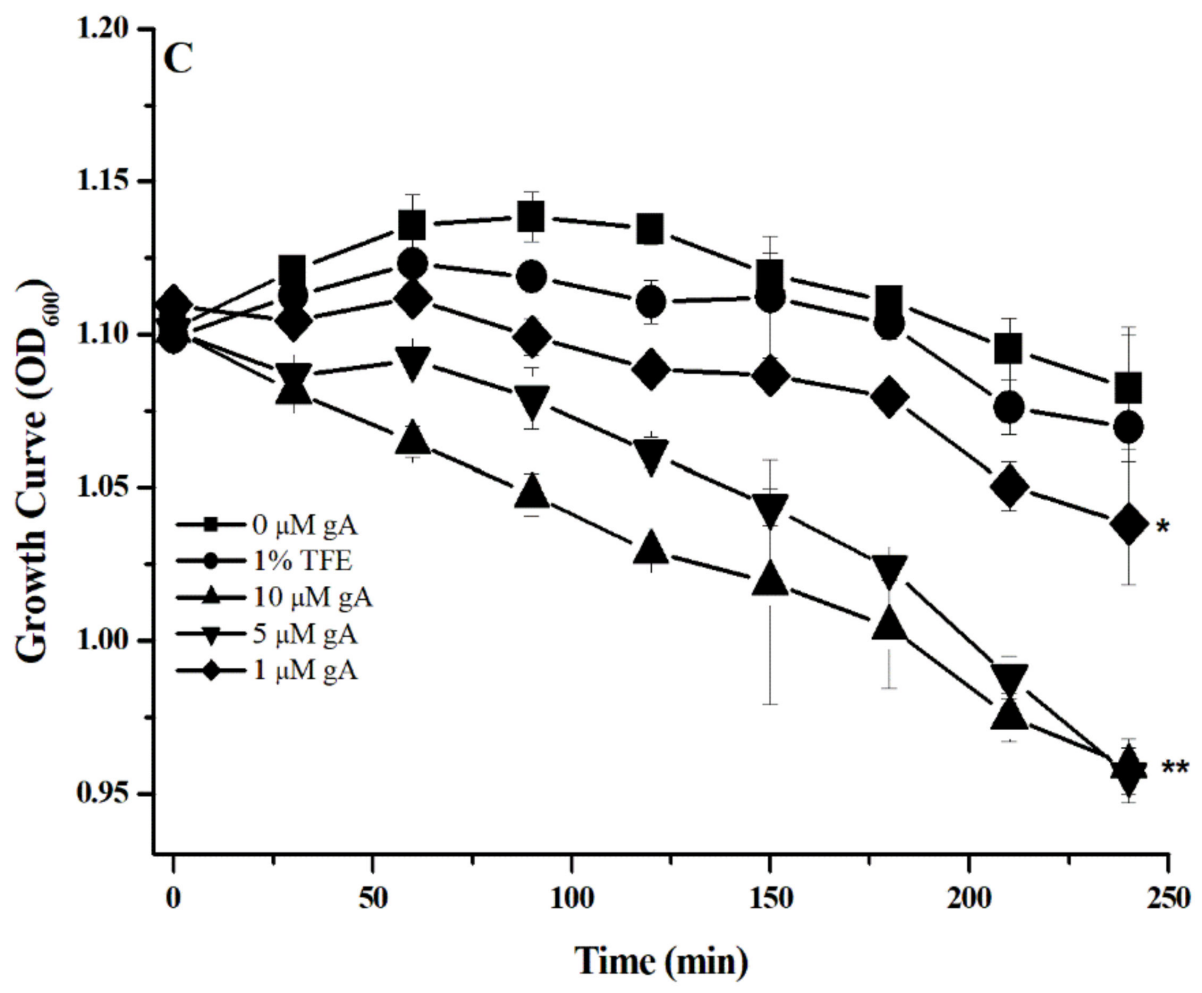
The growth curve of *S. aureus* in the presence of gramicidin A 0, 1, 5, and 10 μM, and 1% TFE without gramicidin A, at the different growth phases, (**A**) lag phase, (**B**) elongation phase, and (**C**) stationary phase, respectively. (*n* = 3, * *p* ≤ 0.05, ** *p* ≤ 0.001, related to 0 μM gA).

We first verified the effect of gA concentration on the growth rate of *S. aureus* at the different bacterial growth phases. Traditionally, the growth phase of bacteria can be classified into lag, exponential and stationary phases [24]. Figure 1A–C shows the growth curve of *S*. *aureus* at the lag phase, exponential phase, and stationary phase in the presence of 0, 1, 5, and 10 μM gA, respectively. For the lag phase (Figure 1A), the inhibition of *S*. *aureus* growth increased with an increase in gA concentration. At the gA concentration ≥ 1 μM, the growth of *S. aureus* was completely inhibited. Figure 1B shows the growth curves for *S. aureus* at the exponential phase in the presence and absence of gA. Bacterial growth was significantly impeded by gA at the concentration ≥ 1 μM. Figure 1C shows the growth curve of *S. aureus* with or without gA treatment at the stationary phase. Similarly, at a concentration ≥ 0.5 μM, the growth of bacteria was completely inhibited. Our results are similar to a previous study that gramicidin A can effectively inhibit the growth of *S. aureus* at all bacterial growth phases [18]. The exponential phase of *S. aureus* was used for the consequent studies.

### 3.2. Verification of the Hydroxyl Free Radical Formation and NAD/NADH Ratio by Gramicidin A

**Figure 2:**
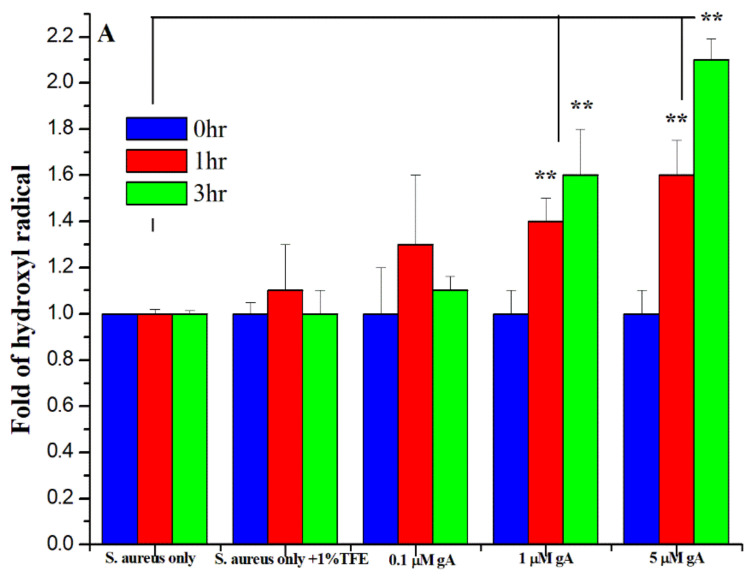

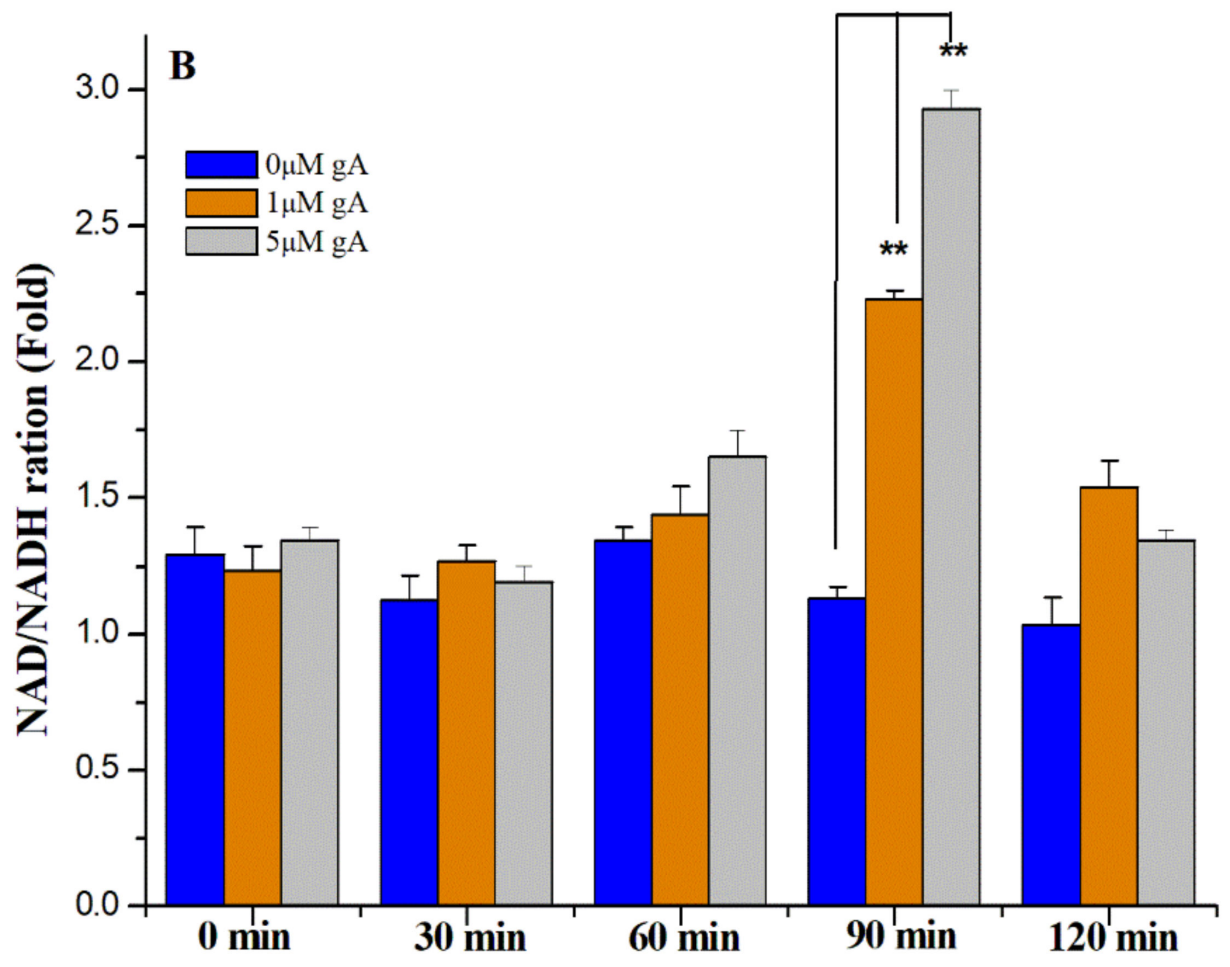
(**A**) Hydroxyl free radical level for *S. aureus* under the treatment of 0, 0.1, 1, and 5 μM of gramicidin A, and (**B**) the NAD^+^/NADH ratio measured for *S. aureus* under the treatment of 0, 1, and 5 μM of gramicidin A and 0, 30, 60, 90, and 120 min at the elongation state. (*n* = 9, ** *p* < 0.001).

Previously, a study has demonstrated that the antimicrobial activity of gramicidin A is associated with the increase of hydroxyl free radical levels and NAD/NADH ratios [18]. We then examined the level of hydroxyl free radicals vs. gA concentration at the exponential phase, as shown in Figure 2A, for 0, 1, and 3 h, respectively. The hydroxyl free radical level was increased with an increase in gA concentration. The levels of hydroxyl free radical at 3 h (green bar in Figure 2A) are almost 1.6-fold (for 1 μM gA) and 2-fold (for 5 μM gA) higher than that of the control (without gA).

The NAD^+^/NADH ratio measured for different gA concentrations is shown in Figure 2B. The NAD^+^/NADH ratio increased with an increase in gA concentration and treatment time. At 90 min, the NAD^+^/NADH ratios for 1 and 5 μM gA are nearly 2.3- and 3-fold higher than that of 0 μM gA, respectively, suggesting that gA can cause an imbalance of NAD^+^/NADH metabolism. The results from hydroxyl free radical levels and NAD^+^/NADH ratios are consistent with the previous study [18].

### 3.3. Effect of Calcium Ion on the Antimicrobial Activity of Gramicidin A

**Figure 3:**
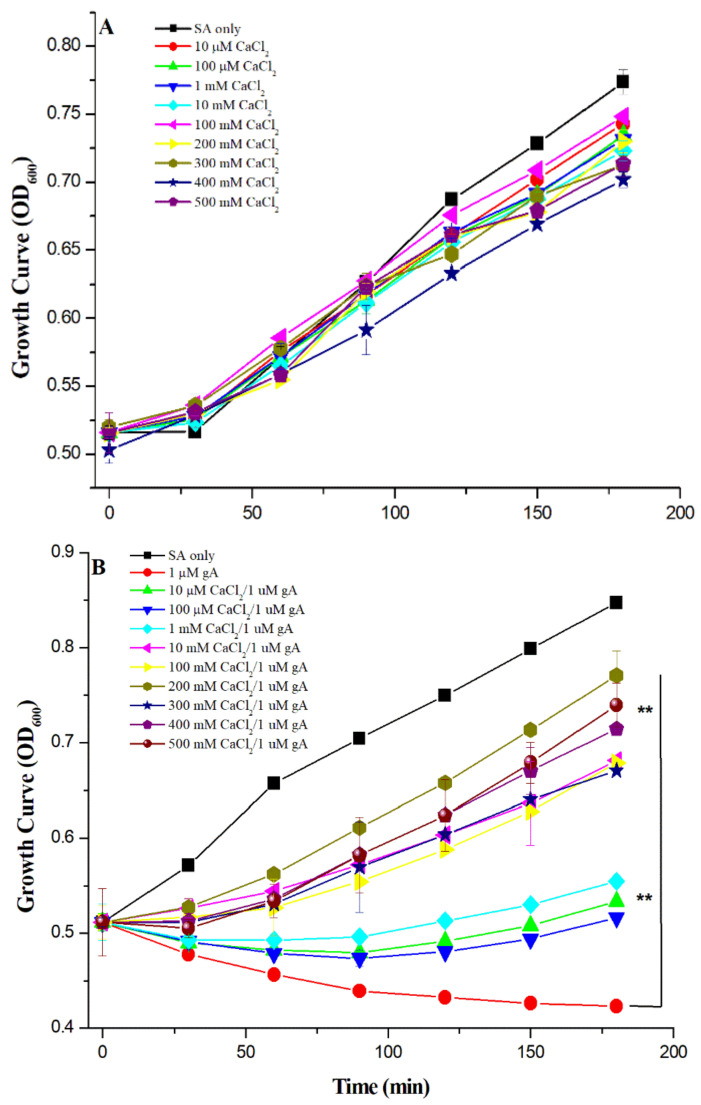

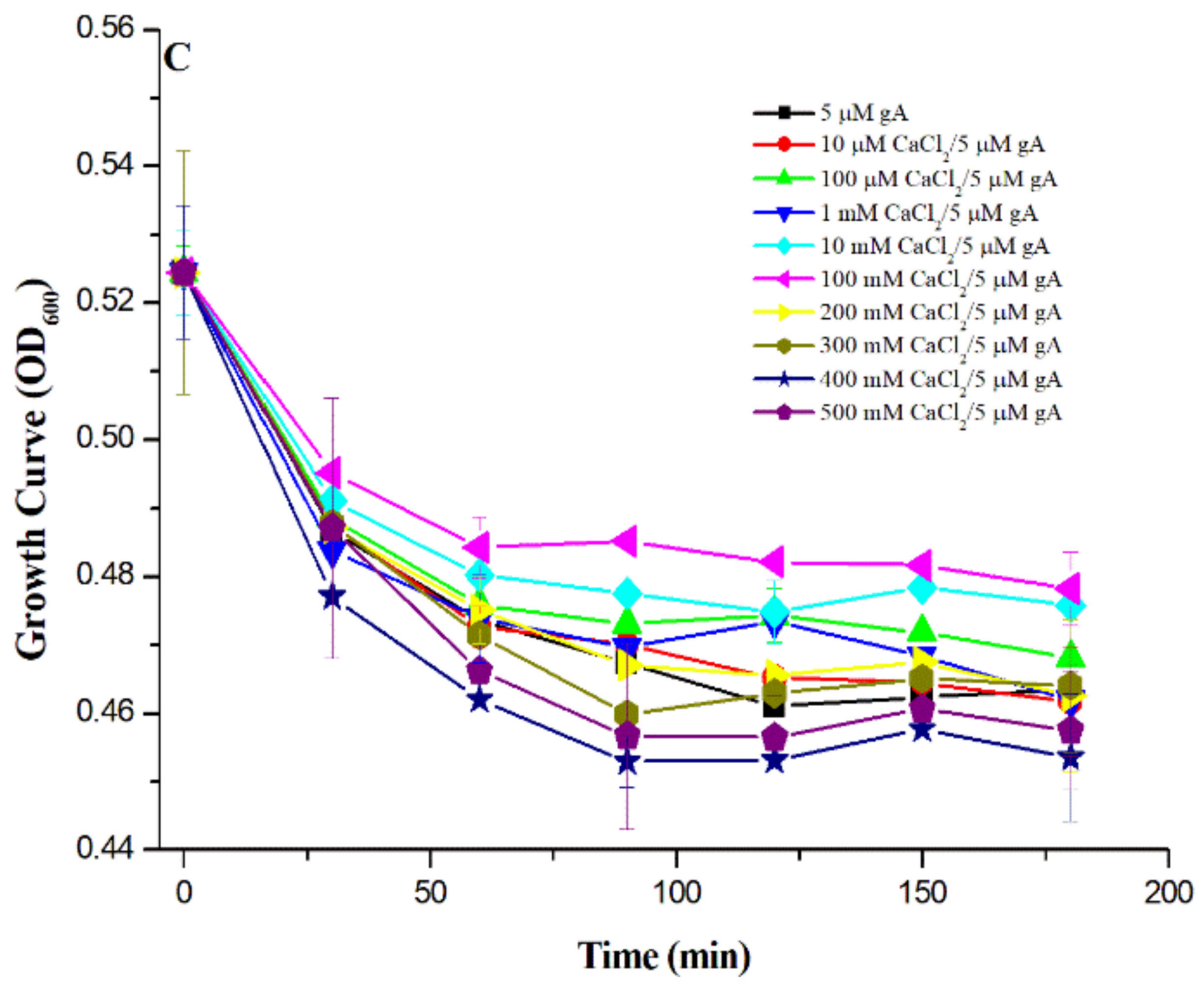
Effects of calcium ions on the growth rate of *S. aureus* in the presence of (**A**) 0–500 mM Ca^2+^ concentrations only, (**B**) 0–500 mM of Ca^2+^ ions and 1 μM gA treatment, and (**C**) 0–500 mM Ca^2+^ and 5 μM gA treatment, respectively. (*n* = 3, ** *p* ≤ 0.001, related to 1 μM gA in (**B**)).

Previous studies have shown that Ca^2+^ ions can block the translocation of monovalent cations across the lipid membrane [22,23]. To illustrate the role of Ca^2+^ ions, we investigated the effect of Ca^2+^ ions on the antimicrobial activity of gramicidin A. Figure 3A shows the growth curve of *S. aureus* in the presence of Ca^2+^ ions without the treatment of gA. The addition of Ca^2+^ ions up to 500 mM did not cause any significant decrease in the growth rate compared to the growth curve of *S. aureus* only, although, when the concentration of Ca^2+^ ions > 300 mM, a slight decrease in the growth rate for *S*. *aureus* was observed. The results indicate that the addition of Ca^2+^ ions does not affect the growth of *S*. *aureus*.

Figure 3B shows the growth rate of *S. aureus* in the presence of 1 μM gA and various concentrations of Ca^2+^ ion. The growth rate of *S. aureus* increased with Ca^2+^ ion concentration. At the concentration of Ca^2+^ ions < 100 mM, the growth rate for *S. aureus* was slightly increased compared to that treated with 1 μM gA only. When the Ca^2+^ concentration ≥ 100 mM, the growth rate of *S. aureus* was significantly increased, indicating that the antimicrobial activity of 1 μM gA can be inhibited by adding a high concentration of Ca^2+^ ions. The growth rate reaches its highest rate at the concentration of 200 μM Ca^2+^ ions. 

Figure 3C shows the survival rate of *S. aureus* in the presence of 5 μM gA and 0–500 mM of Ca^2+^ cations. Unlike the results obtained at 1 μM gA, the antimicrobial activity of gA could not be significantly inhibited by adding Ca^2+^ cations. In comparison with the growth rate of *S. aureus* treated with 5 μM gA, the growth rate of *S. aureus* was slightly increased with an increase in Ca^2+^ ion concentration at the Ca^2+^ ion concentration ≤ 100 mM, whereas the growth rate of *S. aureus* was decreased at the Ca^2+^ ion concentration > 100 mM. Our results suggest that the addition of Ca^2+^ ions cannot inhibit the antimicrobial activity of 5 μM gramicidin A.

### 3.4. Effect of Ca^2+^ Ions on the Hydroxyl Radical Formation

**Figure 4:**
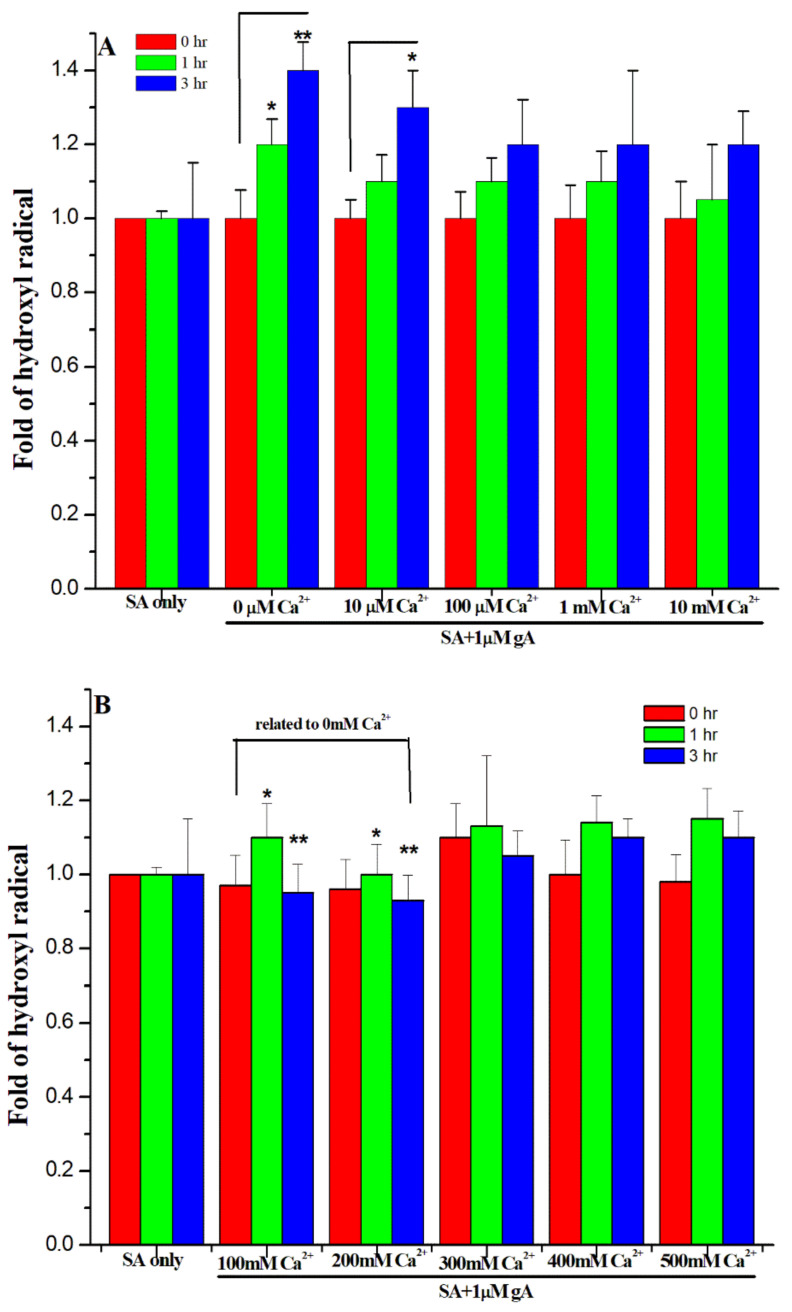

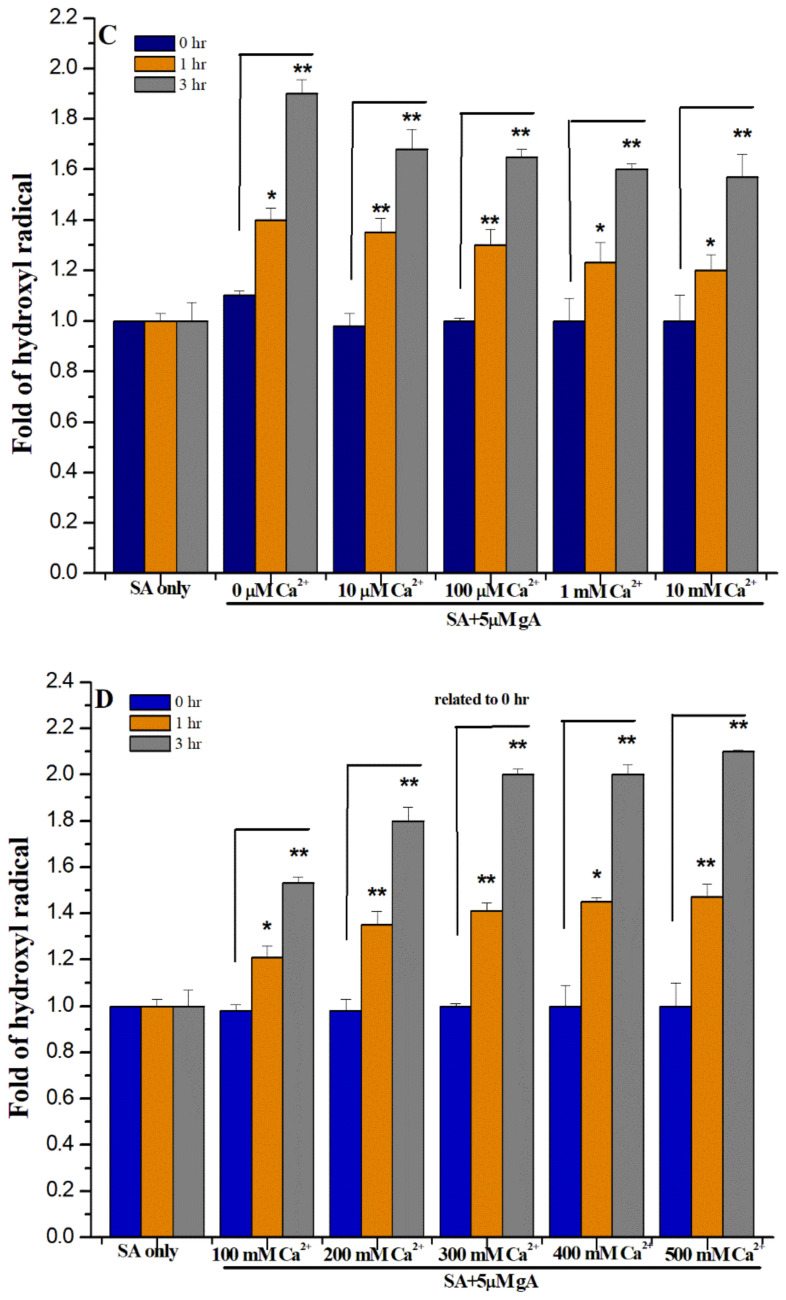
Hydroxyl radical level in the presence of gA and 10 μM–500 mM of gramicidin A, (**A**) 1 μM gA/0–10 mM of Ca^2+^ ions, (**B**) 1 μM gA/100–500 mM of Ca^2+^ ions, (**C**) 5 μM gA/0–10 mM of Ca^2+^ ions, and (**D**) 5 μM gA/100–500 mM of Ca^2+^ ions, respectively. (*n* = 9, ** *p* < 0.001, * *p* < 0.05).

Since the mechanism of gA antimicrobial activity is linked with the formation of hydroxyl radicals [18], we then examined the effect of Ca^2+^ ions on the hydroxyl free radical formation for *S. aureus* in the presence of 1 and 5 μM gramicidin A. Figure 4A,B show the hydroxyl free radical level in the presence of 1 μM gA and Ca^2+^ ion concentration of 0–10 mM and 100–500 mM, respectively. In comparison with the treatment of 1 μM gA only (0 μM of Ca^2+^ in Figure 4A), the hydroxyl free radical level was reduced with an increase of Ca^2+^ ion concentration, indicating that Ca^2+^ ion can reduce the antimicrobial activity of gramicidin A. The hydroxyl free radical level for the treatment of 100 and 200 mM of Ca^2+^ ions at 3 h was 17.8% and 19.8% lower than that with 1 μM gA treatment only, respectively. Our results indicate that high concentrations of Ca^2+^ ions can effectively inhibit the antimicrobial activity of 1 μM gramicidin A.

On the other hand, the effect of Ca^2+^ ions on the hydroxyl free radical level with the treatment of 5 μM gA was not as effective as that with 1 μM gA treatment. In comparison with the treatment of 5 μM gA only (0 μM of Ca^2+^ in Figure 4C), the hydroxyl free radical level was slightly reduced at the concentration of Ca^2+^ ions ≤ 100 mM. At the concentration of Ca^2+^ ions higher than 100 mM, the hydroxyl free radical level was even higher than that with the treatment of 5 μM gA only. Our results suggest that Ca^2+^ ions cannot effectively inhibit the hydroxyl free radical formation induced by 5 μM gramicidin A.

### 3.5. Effect of Ca^2+^ Ions on the NAD^+^/NADH Ratio

**Figure 5:**
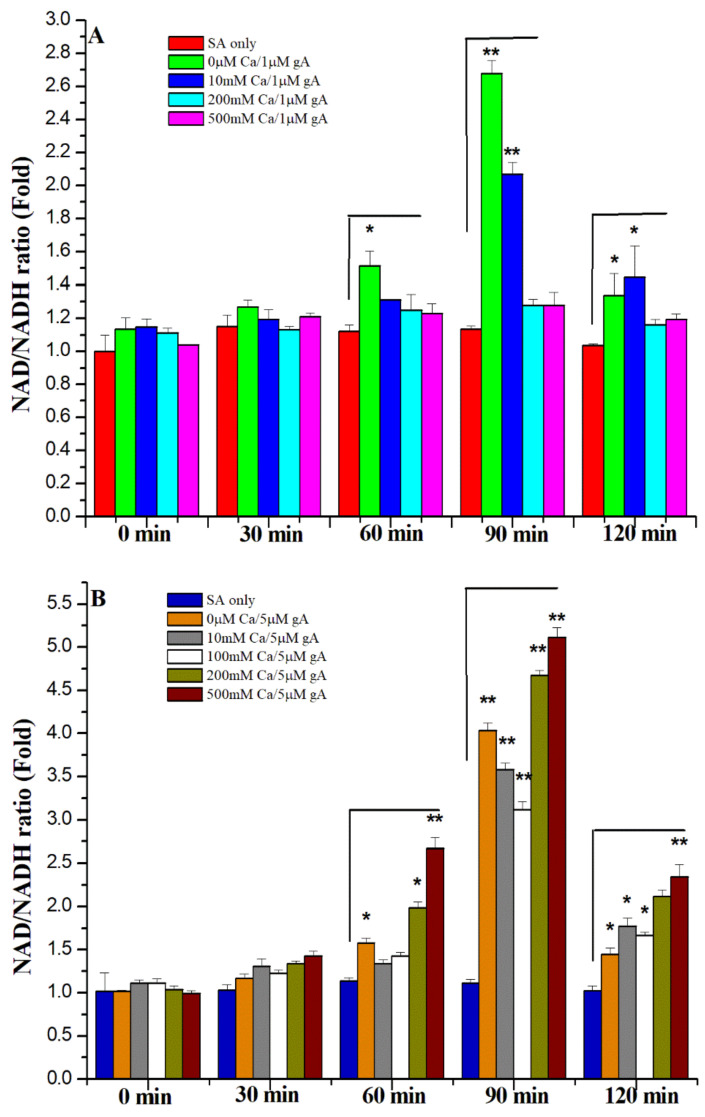
The NAD^+^/NADH ratios for *S. aureus* treated with (**A**) 1 and (**B**) 5 μM gramicidin A and 10, 100, 200, and 500 mM Ca^2+^ ions at 0, 30, 60, 90, and 120 min, respectively. (*n* = 9, ** *p* < 0.001, * *p* < 0.05).

Previously, we showed that hydroxyl radical formation is associated with an imbalance of the NAD^+^/NADH ratio [18]. We examined the NAD^+^/NADH ratio of *S. aureus* in the addition of Ca^2+^ ions. The NDA^+^/NADH ratios were measured with the treatment of 1 and 5 μM gA and Ca^2+^ ion concentrations of 0, 10, 100, 200, and 500 mM for every 30 min from 0 to 2 h. Figure 5A,B shows the Ca^2+^ effects on the NAD^+^/NADH ratios for *S. aureus* with either 1 or 5 μM gramicidin A, respectively. By adding the Ca^2+^ cations, the NAD^+^/NADH ratios decreased with an increase in Ca^2+^ concentration. The NAD^+^/NADH ratio in the presence of 1 μM gA only was 2.6-fold higher than that without the treatment of gA at 90 min (Figure 5A). The NAD^+^/NADH ratios in the presence of either 200 or 500 mM Ca^2+^ ions were similar to that of the control (SA only) at 90 min, suggesting that the addition of a high concentration of Ca^2+^ ion can inhibit the imbalance of NAD^+^/NADH ratios caused by gramicidin A.

The NAD^+^/NADH ratio for the treatment of 5 μM gA was 4.2-fold higher than that of the control (SA only) at 90 min (Figure 5B), indicating that the high concentration of gA can cause a more severe imbalance of NAD^+^/NADH metabolism. Unlike the results for 1 μM gA treatment, the addition of Ca^2+^ ions could not significantly reduce the NAD^+^/NADH ratios to the same level as the control (SA only). At 90 min, the NAD^+^/NADH ratio was 3-fold in the addition of 100 mM Ca^2+^ ions and 5.0-fold for the 500 mM of Ca^2+^ compared to that of the control (SA only). Our results demonstrate that Ca^2+^ ions cannot inhibit the imbalance of NAD^+^/NADH metabolism induced by the 5 μM of gA. This result is consistent with the trends of hydroxyl free radical levels for *S. aureus* treated with 5 μM gramicidin A. 

### 3.6. AFM Images of S. aureus Treated with gA and Ca^2+^ Ions

**Figure 6:**
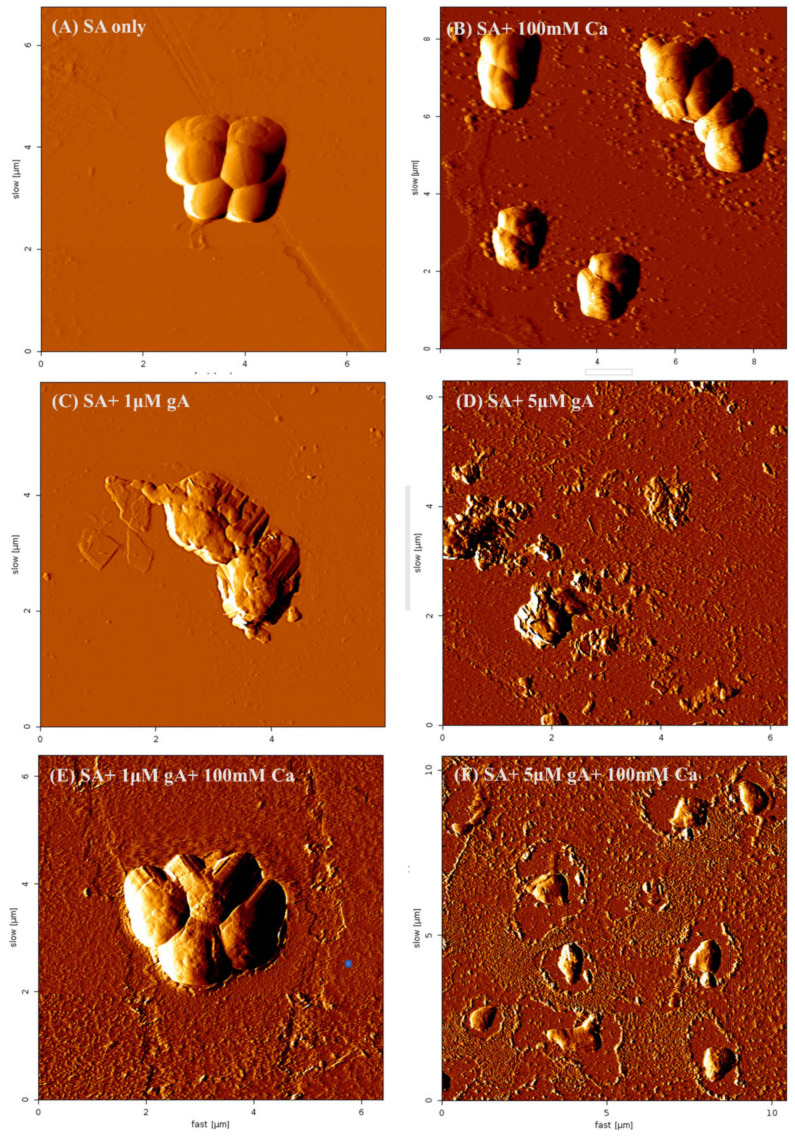
Atomic force microscopic morphologies of *S. aureus* under treatment with gramicidin A and CA^2+^ ions, (**A**) *S. aureus* only without gA, (**B**) with 100 mM Ca^2+^ ions, (**C**) with 1 μM gA, (**D**) with 5 μM gA, (**E**) with 1 μM gA and 100 mM Ca^2+^ ions and (**F**) with 5 μM gA and 100 mM Ca^2+^ ions.

As observed in the previous sections, the Ca^2+^ effects on the antimicrobial activity of gramicidin A were gA concentration-dependent. To further confirm the Ca^2+^ role, AFM microscopy was applied to observe the effect of Ca^2+^ ions on the morphology of *S. aureus*. The morphologies for *S. aureus* under the treatment of 1 and 5 μM gA and various concentrations of Ca^2+^ cations were delineated in Figure 6. The morphologies of *S. aureus* in the absence of gA with or without Ca^2+^ ions were round, smoothed, and aggregated, and the integrity of the membrane surface was maintained (SA only and SA + 100 mM Ca^2+^ in Figure 6A,B, respectively). Following the treatment with either 1 or 5 μM gA (SA + 1 μM A and SA + 5 μM gA in Figure 6C,D, respectively), the morphologies of *S. aureus* were destructive, flattened and lost their membrane integrity compared with the morphologies without the treatment of gA in Figure 6A,B. The bacterial membrane surface further formed a micelle-like shape under the treatment of 5 μM gA (Figure 6D). These results indicate that gA may induce different antimicrobial mechanisms dependent on the concentration.

By adding 100 mM Ca^2+^ ions to the culture medium, the morphologies of *S. aureus* for the treatment of 1 and 5 μM gA (SA + 1 μM gA + 100 mM Ca^2+^ and SA + 5 μM gA + 100 mM Ca^2+^ in Figure 6E,F, respectively) were different. The morphologies of *S. aureus* in the presence of 1 μM gA and 100 mM Ca^2+^ ions were round, smoothed, and clumping to each other similar to the morphologies for *S. aureus* without the treatment of gramicidin A (Figure 6A,B), indicating that Ca^2+^ ions can effectively inhibit the gA-induced bacterial death. Unlike the morphologies that appeared in the presence of 1 μM gA and 100 mM Ca^2+^ ions, the morphologies of *S. aureus* lost the integrity of the membrane surface, round shape, and clumping in the presence of 5 μM gA and 100 mM Ca^2+^ ions, suggesting that Ca^2+^ ions cannot effectively inhibit the antimicrobial activity of high concentration of gA. Our AFM morphological study demonstrates that the antimicrobial mechanism of gramicidin A is dependent on the gA concentration, and the Ca^2+^ effects on antimicrobial activity are correlated with the concentration-dependent mechanism.

## 4. Discussion

Both the hydroxyl free radical formation and dysregulation of the NAD^+^/NADH mechanism have been linked with the antimicrobial activity of gramicidin A [18]. Also, the antimicrobial activity of gramicidin A is highly associated with the conformation of gramicidin A [19,20,21]. Several studies have demonstrated that gramicidin A, only in a channel form, is able to proceed with its antimicrobial activity [19,20,21]. Ca^2+^ ions have been found to block the translocation of alkali cations of gA across membrane lipids and convert gA double helical dimers into helical monomers [21,22,23]. However, the effect of Ca^2+^ ions on the antimicrobial activity of gramicidin A remains unresolved. 

In the present study, we applied biochemical and biophysical methods to study the effect of Ca^2+^ ions on the antimicrobial activity of gramicidin A. In this study, there are two new findings. The first one is that the antimicrobial activity of gramicidin A has two modes. The second finding is that the effects of Ca^2+^ ions on the antimicrobial activity of gramicidin A are dependent on the antimicrobial mechanism via gA concentration. 

The antimicrobial mechanisms of gramicidin A are concentration-dependent. The AFM morphologies for *S. aureus* under the treatment of 1 and 5 μM gA are very different. At low concentrations (gA ≤ 1 μM), the morphologies of *S. aureus* do not show severe membrane destruction and detergent-like shape. Gramicidin A mainly induces hydroxyl free radical formation and NADH metabolic imbalance. This is possible through the translocation of cations across the cell membranes.

At high concentrations (5 μM gA), the morphologies of *S. aureus* tend to be more destructive and form a detergent-like shape. The formation of detergent-like morphology may indicate that the higher concentration of gramicidin A may aggregate on the membrane surface and disrupt the membrane lipid composition more severely than the lower concentration of gramicidin A. Hence, gramicidin A mainly causes severe cell membrane permeabilization and disrupts the membrane integrity with a level-up of hydroxyl free radicals and NAD^+^/NADH ratios. The former corresponds to the channel function, which is more likely associated with the disruption of cell membrane composition by the aggregation of antimicrobial peptides on the membrane surface.

The effect of Ca^2+^ ions on the antimicrobial activity of gramicidin A is dependent on the concentration of gramicidin A. At the concentration of gramicidin A ≤ 1 μM, Ca^2+^ ions can significantly inhibit the antimicrobial activity of gramicidin A. The reduction of antimicrobial activity is dependent on the Ca^2+^ ion concentration. The high Ca^2+^ concentration inhibits the antimicrobial activity more effectively than the low Ca^2+^ concentration. The inhibition of antimicrobial activity is well correlated with the reduction of hydroxyl free radical formation and NAD^+^/NADH metabolic imbalance [18]. One of the possible reasons for the reduction of antimicrobial activity of gramicidin A may be due to the block of translocation of cations, possibly H^+^, by Ca^2+^ ions [22,23]. 

Unlike the inhibitory effect of Ca^2+^ ions on 1 μM gA, the inhibitory ability of Ca^2+^ ions on the antimicrobial activity of 5 μM gramicidin A was almost abolished. The addition of Ca^2+^ ions cannot significantly rescue the growth rate of *S. aureus* and inhibit the hydroxyl free radical formation and the imbalance of NADH metabolism under the treatment of 5 μM gA, although a slight increase in the survival rate of *S. aureus* was observed for the Ca^2+^ ion concentration ≤ 100 mM. Two possible reasons may account for this loss of the inhibitory ability of Ca^2+^ ions on the antimicrobial activity of 5 μM gramicidin A. One is that a much higher concentration of Ca^2+^ ions is needed for complete inhibition of the antimicrobial activity of 5 μM gramicidin A. The other possibility is that 5 μM gramicidin A undergoes a different antimicrobial mechanism. Taking the antimicrobial mechanism into account, we suggest that undergoing a different antimicrobial mechanism is the most likely reason for abolishing the Ca^2+^ inhibitory effect on the antimicrobial activity of 5 μM gramicidin A. Similar observations are also reported by a previous study [18]. Hence, the inhibition of Ca^2+^ ions on the antimicrobial activity becomes ineffective under the treatment of 5 μM gramicidin A. Furthermore, it is worth notifying that when the concentration of Ca^2+^ ions > 100 mM, Ca^2+^ ions can enhance the antimicrobial activity, as the growth rate, hydroxyl free radical level, and NAD^+^/NADH ratios are all increased compared to those of 5 μM gA only.

In conclusion, using Ca^2+^ ions, we disclosed that the antimicrobial mechanism of gramicidin A is dependent on the gA concentration. At lower concentrations (≤ 1 μM), the antimicrobial mechanism of gramicidin A is mainly linked with the imbalance of NADH metabolism and the formation of free radicals caused by the translocation of cations across the bacterial membrane lipids, while at higher concentrations (≥5 μM), gramicidin A acts like a detergent and cause the disruption and destruction of bacterial membrane lipids. Hence, Ca^2+^ ions can only significantly inhibit the gA antimicrobial activity at the low concentration of gramicidin A (1 μM) by blocking the translocation of cations across the bacterial membrane lipids.

## Data Availability

The data present in the current study are available from the corresponding author upon reasonable request.
